# Placental ACE2 Expression: A Possible Pathogenetic Mechanism for Infantile Hemangiomas

**DOI:** 10.3390/dermatopathology11030020

**Published:** 2024-07-11

**Authors:** Aurora De Marco, Gerardo Cazzato, Rosalba Maggialetti, Giuseppe Ingravallo, Margherita Fanelli, Antonella Vimercati, Ettore Cicinelli, Nicola Laforgia, Iria Neri, Ernesto Bonifazi, Domenico Bonamonte

**Affiliations:** 1 Section of Dermatology, Department of Precision and Regenerative Medicine and Jonian Area, University “Aldo Moro” of Bari, 70121 Bari, Italy; r.maggialetti2@studenti.uniba.it (R.M.); domenico.bonamonte@uniba.it (D.B.); 2 Section of Pathology, Department of Precision and Regenerative Medicine and Jonian Area, University “Aldo Moro” of Bari, 70121 Bari, Italy; giuseppe.ingravallo@uniba.it (G.I.); margherita.fanelli@uniba.it (M.F.); 3 Section of Gynecology and Obstetrics, Interdisciplinary Department of Medicine, University of Bari “Aldo Moro”, 70124 Bari, Italy; antonella.vimercati@uniba.it (A.V.); ettore.cicinelli@uniba.it (E.C.); 4 Section of Neonatology and NICU, Interdisciplinary Department of Medicine, University of Bari “Aldo Moro”, 70124 Bari, Italy; nicola.laforgia@uniba.it; 5 Dermatology Unit, IRCCS Azienda Ospedaliero Universitaria Bologna, University of Bologna, 40126 Bologna, Italy; iria.neri@unibo.it; 6 Independent Researcher, 70124 Bari, Italy; e.bonifazi@ejpd.com

**Keywords:** infantile hemangiomas, ACE2, renin-angiotensin-system, placental hypoxia

## Abstract

ACE2 is a mono-carboxypeptidase with remarkable vasculo-protective properties, and its expression in the human placenta plays a central role in blood pressure homeostasis and fetal perfusion. Therefore, an alteration in the placental expression of ACE2 could be responsible for reduced placental perfusion and infantile hemangioma (IH) development. Study placentae were collected from patients affected by IHs who were referred to our Dermatology Clinic from 2016 to 2022, while control placentae were randomly collected while matching cases for gestational age. Immunohistochemical investigations were performed with a recombinant anti-ACE2 rabbit monoclonal antibody. A total of 47 placentae were examined, including 20 study placentae and 27 control ones. The mean placental weight was significantly lower in the study group (380.6 g vs. 502.3 g; *p* = 0.005), while subclinical chorioamnionitis occurred more frequently in the study group (20% vs. 0%, *p* = 0.03). The mean ACE2 expression was dramatically lower in the study group (χ2 = 42.1 *p* < 0.001), and the mean placental weight was significantly lower when ACE2 was not expressed compared to the 25–75% and >75% classes of expression (*p* < 0.05). This study demonstrated that ACE2, as a marker for tissue hypoxia, is dramatically hypo-expressed in placentae belonging to mothers who delivered one or more babies with IH compared to the controls.

## 1. Introduction

The renin–angiotensin system (RAS) is currently considered to be made up of two arms, represented by angiotensin-converting enzyme (ACE)/angiotensin (Ang)-II with vasoconstricting properties and ACE2/Ang-(1-7) with vasodilating, anti-thrombotic, and anti-inflammatory properties [1]. ACE2 is indeed a mono-carboxypeptidase known for hydrolyzing Ang-I and Ang-II in Ang-(1-9) and Ang-(1-7), respectively [1]. Therefore, decreasing ACE2 activity may induce a pro-thrombotic state due to the increased levels of Ang-II and decreased levels of Ang-(1-7) [1].

ACE2 is widely expressed in the human body and especially in the kidneys, followed by the lungs, testis, heart, gastrointestinal tract, liver, uterus, and placenta [2]. However, during pregnancy, placental ACE2 expression is even greater than that registered in the kidneys, thus suggesting that ACE2 may play a central role in pregnancy homeostasis [2,3].

Interestingly, even though a sort of resistance to Ang-II has traditionally been described during pregnancy in order to explain why blood pressure does not increase in healthy pregnant women despite the higher levels of Ang-II, more recent studies have demonstrated that the ACE2/Ang-(1-7) axis may be responsible for this mechanism [3,4].

Therefore, it is not surprising that an imbalance in the Ang-II and Ang-(1–7) pathways that may favor Ang-II has been reported in pregnancies with preeclampsia (PE) and fetal growth restriction (FGR) [3]. Furthermore, a significant negative correlation between maternal blood pressure and Ang-(1–7) levels has indeed been demonstrated; in addition, human FGR placentae, similarly to PE placentae, seem to display reduced ACE2 expression [3].

Moreover, in ACE2 knockout mice, the absence of ACE2 is associated with lower fetal weight, increased levels of placental hypoxic markers, and lower oxygen placental levels, thus directly correlating ACE2 hypo-expression with placental hypoxia [5,6].

**Figure 1:**
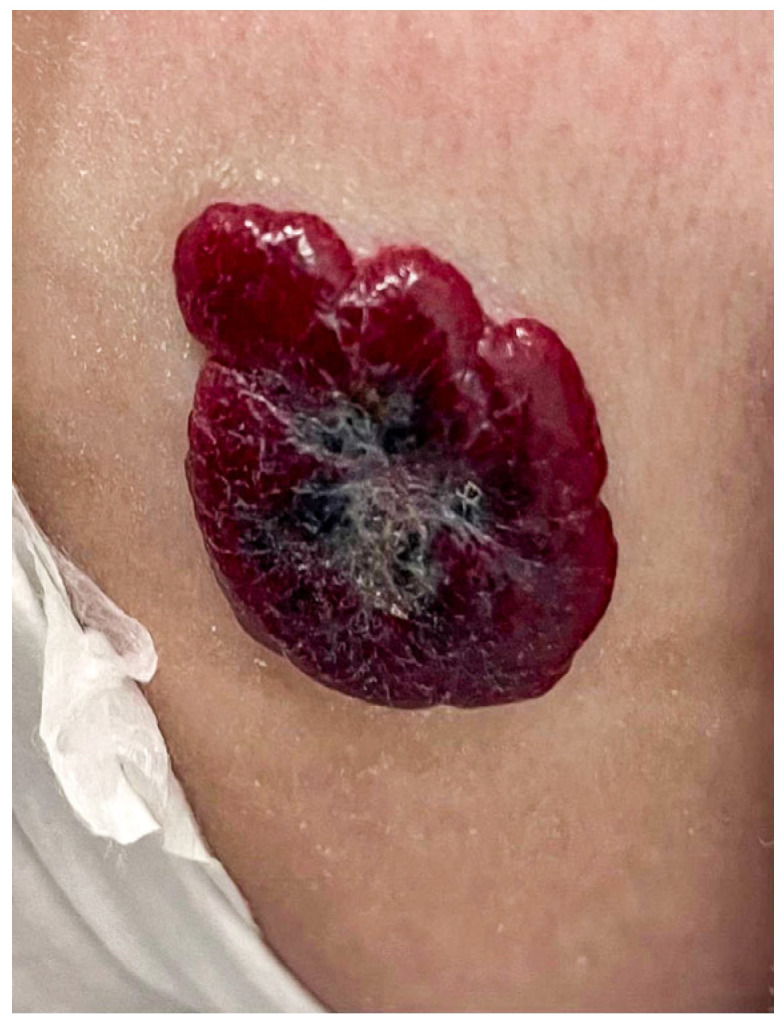
An example of a superficial plaque-like “strawberry-red” infantile hemangioma in a 4-month-old boy.

On the other hand, infantile hemangiomas (IHs), which are traditionally associated with placental hypoxia, are the most common benign pediatric vascular tumors, with a prevalence of 4.5% under the age of nine months [7,8]. Clinically, preferred anatomic locations are represented by the head/neck, trunk, and extremities, with rare cases of visceral involvement, such as in the lungs, liver, gastrointestinal tract, and brain [8]. Furthermore, IHs are characterized by different growth phases, from the initial macules/patches to plaques that can undergo involution [9,10]. Figure 1 shows an example of an IH in the superficial plaque stage.

**Figure 2:**
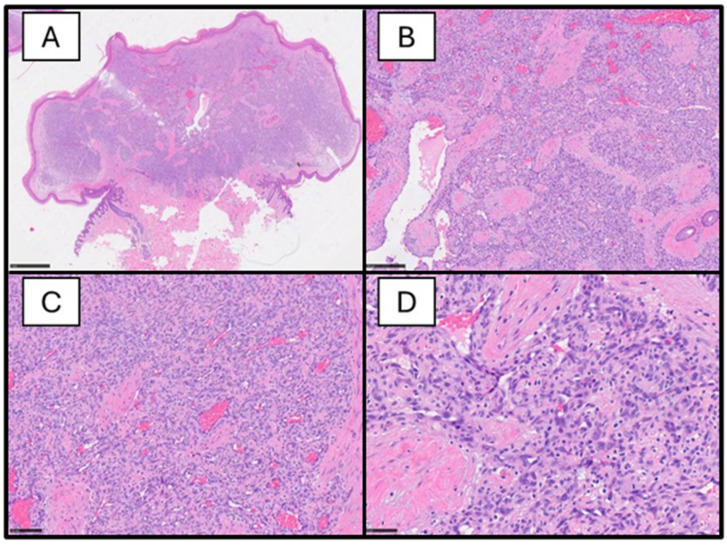
(**A**) Histological photomicrograph showing a panoramic view of an example of infantile hemangioma; note the lobulated proliferation (H&E, original magnification: 2.5×). (**B**) Histological preparation showing a higher magnification of a field of the previous picture; note the closely packed capillaries with some foci of hemorrhage and fibrosis (H&E, original magnification: 10×. (**C**) Higher magnification showing the previous features with more details (H&E, original magnification: 20×). (**D**) Histological picture showing some mitotic figures in the vascular proliferation, which is a typical feature of IH (H&E, original magnification: 40×).

From a histological point of view, the lesions are characterized by the lobular growth of closely packed capillaries lined by plump endothelial cells, sometimes with numerous mitoses. An example of the histological features of an IH is reported in Figure 2.

Although their pathogenesis has not yet been completely clarified, the embolization of placental stem cells, the hypoxic placental trigger, and the RAS dysregulation are currently considered the most reasonable pathogenetic theories for IHs [11].

Therefore, as a correlation between placental ACE2 expression and placental hypoxia has been postulated, the present study aims to link placental ACE2 expression to the possible development of IHs in newborns.

## 2. Materials and Methods

As in normal clinical practice, all placentae of patients who give birth in our hospital are histologically examined and subsequentially archived, the study placentae were selected from patients who delivered in our hospital and were referred to our Dermatology Clinic from 2016 to 2022 for the presence of IH. Dichorionic twins and women who tested positive for severe acute respiratory syndrome coronavirus 2 (SARS-CoV-2) at the time of delivery were excluded in order to avoid bias in the case of a single twin with IH and in fetal perfusion, respectively [12].

Control placentae from SARS-CoV-2-negative women were randomly collected from our Pathology Institute while matching cases for gestational age (GA) (matching confirmed via the Student *t*-test). Women were subsequentially contacted, and only those who did not report the development of IHs in their babies were enrolled in the control group.

Due to the retrospective nature of the present work, no other clinical parameters (e.g., hypertension, diabetes, smoking habits, etc.) were available and, therefore, used for case–control matching.

Morphological and histological descriptions of all placentae from both groups were already available in our Pathology Institute. All placentae were fixed in formalin buffered at 10%, weighed, sampled, and examined along the cut surface. All samples underwent routine treatment (inclusion, 5 µm sectioning, and hematoxylin–eosin staining), and they were examined with an Olympus BX-51 optical microscope equipped with an Olympus DP80 image acquisition system. Further immunohistochemical investigations were conducted with recombinant anti-ACE2 rabbit monoclonal antibody [EPR4435] (ab108252) at a 1:500 dilution. The preparations were examined by two dermatopathologists (G.C. and G.I.), and if they disagreed, a third one from the same institute was asked to discuss the case and examine it. A positive control for ACE2 antibody was performed with kidney sections.

Therefore, a total of 47 placentae were examined, belonging to 46 different patients, thus including 20 women who delivered one or more babies with IH (study group) and 26 women who delivered babies without IH (control group).

ACE2 expression was assessed using manual counting for scoring as follows: “0” for negative (<1% of stained cells), “1” for low (≥1% to <25%), “2” for intermediate (25% to <75%), and “3” for high (≥75%).

Both groups were described and compared in terms of the mean placental weight, ACE2 expression, prematurity, twinning, fetal gender, and placental histological features.

A two-sample *t*-test was used to compare the mean placental weight in the study group with that registered in the control group (normal distribution of placental weights verified by using the Kolmogorov–Smirnov test).

A χ2 test has been carried out to compare the distribution of ACE2 expression between the two groups, while the Cochrane–Armitage (C-A) trend test was used to evaluate the presence of a significant trend. A χ2 test was performed to compare all other pregnancy parameters and placental histological features between the two groups, while the Fisher exact test was used for frequencies less than 5. Analysis of variance, followed by Tukey’s HSD post hoc test, was performed to compare placental weights among classes of ACE2 expression.

## 3. Results

A total of 47 placentae were examined, belonging to 46 different patients. Therefore, two groups were established, thus including 20 women who delivered one or more babies with IH (study group) and 26 women who delivered babies without IH (control group).

The age of the patients in the study group ranged from 26 to 43 years (mean age: 35.05), while the controls turned out to be significantly younger (age range: 20–37; mean age: 26.77; *p* < 0.001).

Prematurity was registered in 16 cases out of 20 in the study group, without any differences from the controls (80% vs. 78%; *p*-value 0.85), as both groups were matched for GA (34.7 vs. 35.6; *p*-value 0.21). Moreover, twins were reported in two cases out of 20 in the study group (monochorionic) and in two cases out of 27 in the control group (monochorionic and dichorionic), without any statistically significant differences (*p* = 0.75) (Table 1).

As for the study group, IHs were more frequently mixed or profound (13 cases out of 20; 65%), while superficial IHs developed in only seven patients (35%).

The mean placental weight was significantly lower in the study group (380.6 g) than in the control group (502.3 g; *p* = 0.005), while, according to the histological findings, subclinical chorioamnionitis occurred more frequently in the study group than in the controls (20% vs. 0%, *p* = 0.03), without any differences in the percentages of the other histological parameters (Table 1).

**Figure 3:**
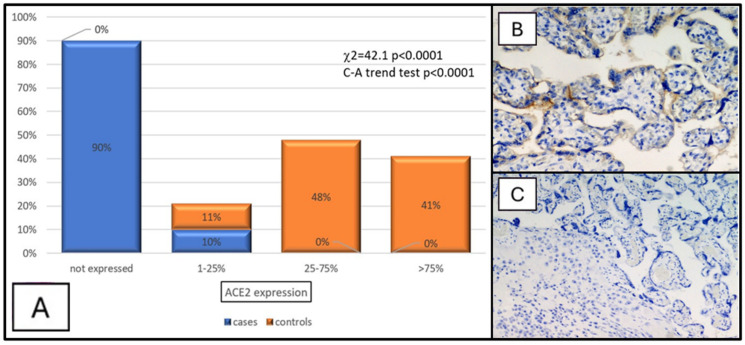
(**A**) Graphical ACE2 expression in the cases and controls. (**B**) Photomicrograph showing positivity (brown signal) in decidual cells for ACE2 monoclonal antibody of a patient in the control group (immunohistochemistry for ACE2, original magnification: 40×). (**C**) Photomicrograph showing almost complete negativity in decidual cells for ACE2 of a patient from the study group (immunohistochemistry for ACE2, original magnification: 20×).

**Figure 4:**
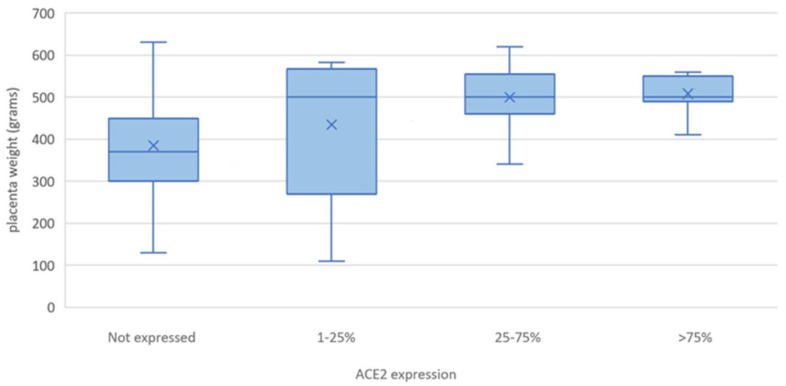
Placental weight in relation to ACE2 expression. Means are represented by X.

The mean ACE2 expression turned out to be dramatically lower in the study group than in the control group (χ2 = 42.1, *p* < 0.001, C-A trend test: *p* < 0.0001) (Figure 3A–C), and the mean placental weight was significantly lower when ACE2 was not expressed than in the 25–75% and >75% classes of expression (*p* < 0.05) when placentae were classified in relation to ACE2 expression (F = 3.2, *p* = 0.03) (Figure 4).

## 4. Discussion

IHs are the most common benign pediatric vascular tumors, with a prevalence of 4.5% under the age of nine months [7,8]. Although their pathogenesis has not yet been completely clarified, three main pathogenetic theories for IHs are currently accepted: (1) the embolization of placental stem cells, (2) the maternal hypoxic pathogenetic trigger, and (3) the hyperactivation of the RAS [8]. If, on one hand, both IHs and placental tissue indeed share common vascular markers, thus supporting the idea that IHs could originate from the embolization of placental stem cells, on the other hand, a certain pathogenetic role of the RAS cannot be denied. As a matter of fact, higher levels of renin and Ang-II are demonstrated both in pregnant women and in newborns, with a decreasing pattern that reflects IH involution [8,9,10].

Moreover, the idea that placental hypoxic events may lead to the development of IHs strongly correlates with the clinical evidence that several maternal and/or fetal conditions predisposing one to hypoxia are also common risk factors for IHs [7,13,14,15,16]. Many different possible pregnancy complications, such as maternal vaginal bleeding during the first trimester, PE, and placenta previa, have indeed been widely described as common risk factors for IHs [7,8,13,14,15,16]. Moreover, it has been demonstrated that glucose transporter-1 (GLUT-1), the most representative marker for IHs, is a downstream target of hypoxia-inducible factor-1-alpha (HIF-1α), along with vascular endothelial growth factor (VEGF), so that several angiogenetic factors that are upregulated by HIF-1α and associated with angiogenesis under hypoxic conditions are more strongly expressed in IHs than in the surrounding normal endothelial tissue [7,17,18,19].

Lately, on the other hand, scientific attention to ACE2 has increased, as it appears to be a SARS-CoV-2 binding receptor on cell membranes, and it is currently under investigation in oncology for its antiangiogenetic properties, which are mainly displayed throughout VEGF inhibition [19,20].

Interestingly, Wang et al. demonstrated that ACE2 is less expressed in IH cell culture and biopsy tissue than in normal endothelial tissue, as opposed to HIF-1α, thus correlating ACE2 hypo-expression and RAS dysregulation with both IH development and tissue hypoxia [21].

In our study, ACE2 turned out to be dramatically hypo-expressed in all placentae belonging to mothers who delivered one or more babies with IHs compared to the controls (χ2 = 42.1 *p* < 0.001, C-A trend test *p* < 0.0001). These data seem to strongly support the hypoxic pathogenetic theory for IHs, as a reduction in ACE2 expression could have led to the upregulation of both the ACE/Ang-II axis and the VEGF pathway, thus causing placental hypoxia and, possibly, fetal IH development [19,20].

Interestingly, in order to demonstrate the hypoxic pathogenetic theory for IHs, different studies have focused on placental histological abnormalities [22,23]. In particular, López Gutiérrez et al. demonstrated an increased incidence of ischemic infarction in the study placentae, while Colonna et al. pointed out that hypoxia-induced histological alterations occurred more frequently in placentae belonging to women who delivered one or more babies with IHs than in controls [21,22]. However, even if histological features may be strongly indicative of an hypoxic insult, they are often difficult to quantify and compare. On the other hand, an immunohistochemical marker for hypoxia, such as ACE2, may allow more precise investigation, even for cases in which histological alterations may be mild or completely absent.

In our study, indeed, despite a strong difference in ACE2 expression between the two groups, no other relevant differences were found in all examined histological parameters, with the exception of histological chorioamnionitis, which occurred more often in the study group (20% vs. 0%, *p* = 0.03). Interestingly, chorioamnionitis and intraamniotic infections have already been correlated in the literature with the upregulation of VEGF signaling at the maternal–fetal interface; thus, in the present study, placentae belonging to women who delivered one or more babies with IHs displayed a higher rate of chorioamnionitis and a remarkable hypo-expression of ACE2, with both indicating an increased activation of the VEGF pathway, as is commonly found in hypoxic settings [23].

However, as ACE2 is a central part of the RAS, its hypo-expression in the study placentae pathogenetically links the development of IHs not only to placental hypoxia but also to a certain degree of RAS dysregulation. Moreover, as ACE2 hypo-expression has already been demonstrated in IH tissue, the present study also highlights that placental tissue may share common histochemical features with IHs, as suggested by the pathogenetic theory of embolization [7,21].

As for the mean placental weight in the study group, it appeared to be lower than that of the controls, with a certain correlation with ACE2 hypo-expression. Interestingly, low placental weight has been already associated with an increased risk of stillbirth and FGR, thus suggesting a possible correlation of ACE2 hypo-expression with both IHs and severe placental abnormalities [24,25]. In the end, the women from the study group turned out to be significantly older than the controls (mean age: 35.05 vs. 26.77; *p* < 0.001), thus not only supporting previous findings regarding advanced maternal age as a possible risk factor for IH but also suggesting that an older maternal age could be related to altered placental perfusion, as demonstrated by ACE2 hypo-expression [7].

## 5. Conclusions

The present study suggests that ACE2, as a marker for tissue hypoxia, is dramatically hypo-expressed in placentae belonging to mothers who delivered one or more babies with IHs compared to controls. Interestingly, as ACE2 is a central enzyme of the RAS, regulating vascular perfusion and showing lower levels of histochemical expression both in the study placentae and in IH biopsy tissue [21], the present study may offer a different point of view on IH pathogenesis, suggesting a new possible unifying pathogenetic vision.

## Figures and Tables

**Table 1:** Characteristics of pregnancy and placental findings in both the study and control groups. The means were compared using an unpaired *t*-test, percentages were compared using a χ2 test, and the Fisher exact test was used for frequencies less than 5.

Characteristics	Study Group	Control Group	*p*-Value
Placental weight, mean (s.d.)	380.6 (167.3)	502.3 (64)	0.005
Gestational age, mean (s.d.)	34.7 (2.7)	35.6 (1.1)	0.21
Prematurity N (%)	16 (80%)	21 (78%)	0.85
Twins N (%)	2 (10%)	2 (7.4%)	0.57
Maternal malperfusion N (%)	13 (65%)	11 (40.7%)	0.1
Decidual arteriopathy N (%)	4 (20%)	2 (7.4%)	0.2
Fetal malperfusion N (%)	5 (25%)	5 (18.5%)	0.6
Deciduitis N (%)	2 (10%)	0	0.17
Intervillous fibrin deposition N (%)	2 (10%)	4 (14.8%)	0.49
Villous hypervascularity N (%)	4 (20%)	5 (18.5%)	0.59
Fetal thrombotic vasculopathy N (%)	2 (10%)	0	0.17
Syncytial knots N (%)	7 (35%)	13 (48.2%)	0.37
Chorioamnionitis N (%)	4 (20%)	0	0.03

## Data Availability

The data presented in this study are available from the corresponding author upon request due to privacy.
